# Agreement and Diagnostic Accuracy of New Linear Deflection Oscillometry and Doppler Devices for Hypotension Detection Compared to Invasive Blood Pressure in Anesthetized Dogs

**DOI:** 10.3390/vetsci12020116

**Published:** 2025-02-02

**Authors:** Matheus M. Mantovani, Any C. A. Costa, Mayara T. de Lima, Luis F. N. dos Santos, Kimberly F. Silva, Alessandro R. de C. Martins, Adan W. M. Navarro, Renata S. Akabane, Denise T. Fantoni

**Affiliations:** 1 Faculty of Veterinary Medicine and Animal Science, Federal University of Uberlândia, Uberlândia 38410-337, Brazil; any.costa@ufu.br (A.C.A.C.); kimberly.silva@ufu.br (K.F.S.); 2 UFAPE Veterinary Intensive Care Unit, São Paulo 01428-000, Brazil; mayara@intercursos.com.br (M.T.d.L.); alessandromartins@intercursos.com.br (A.R.d.C.M.); willnavarro2008@gmail.com (A.W.M.N.); renataakabane@gmail.com (R.S.A.); 3 Department of Veterinary Clinical Sciences, College of Veterinary Medicine, Purdue University, West Lafayette, IN 47907, USA; dossantos@purdue.edu; 4 Department of Surgery, Faculty of Veterinary Medicine and Animal Science, University of São Paulo, São Paulo 05508-270, Brazil; dfantoni@usp.br

**Keywords:** pulse wave, hemodynamic state, high-definition oscillometric, anesthesia

## Abstract

Blood pressure (BP) monitoring is essential during anesthesia to maintain cardiovascular stability and detect hypotension. This study evaluated the agreement and diagnostic accuracy of linear deflection oscillometry (LDO) and Doppler compared to invasive blood pressure (IBP) in anesthetized dogs. Eleven healthy dogs were anesthetized, and BP measurements were taken using LDO, Doppler, and IBP methods under normotensive and hypotensive conditions. The LDO device demonstrated superior agreement, assessed using Bland–Altman analysis, with IBP, especially in hypotensive conditions, compared to the Doppler method. LDO showed bias and standard deviation in the hypotensive state, with a mean and systolic arterial pressure (MAP and SAP) of −5.1 ± 7.9 and −5.6 ± 12.5 mmHg, respectively. Conversely, Doppler measurements tended to overestimate SAP during hypotension, presenting a bias of −13 ± 15.45 mmHg. The LDO achieved an area under the curve (AUC) of 0.809 for hypotension detection, with an MAP cutoff of ≤72 mmHg (sensitivity: 90%, specificity: 63%). Meanwhile, the best threshold for Doppler measurements was an AUC of 0.798, SAP ≤ 100 mmHg (sensitivity: 77.8%, specificity: 81.8%). These results indicate that LDO is a reliable method for hypotension detection in anesthetized dogs, with potential applications for real-time monitoring. In contrast, the Doppler method may help confirm hypotension diagnoses.

## Simple Summary

Monitoring blood pressure during anesthesia is crucial for maintaining the health and safety of dogs, especially for detecting hypotension, which can lead to serious complications if not managed promptly. The objective of this study was to compare the concordance between two non-invasive methods—an advanced oscillometry device and a Doppler ultrasound device—and the invasive method through the cannulation of a peripheral artery for blood pressure measurement in anesthetized dogs. By testing these methods on anesthetized dogs, we found that the oscillometry device provided more accurate results than the Doppler, especially in identifying hypotension. This finding suggests that the oscillometry device could be a reliable, non-invasive tool for veterinary professionals to monitor blood pressure more effectively during surgery, enhancing patient care and reducing the risks associated with anesthesia. This advancement in non-invasive monitoring could make it easier to detect and respond to changes in a dog’s blood pressure, potentially improving outcomes in veterinary surgeries.

## 1. Introduction

Arterial blood pressure (BP) is defined as the force exerted by circulating blood against the walls of the arteries and is commonly classified into three components: systolic arterial BP (SAP), mean arterial BP (MAP), and diastolic BP (DAP) [1]. SAP reflects the maximum pressure in the arteries during ventricular contraction, indicating the force of blood ejected by the heart. DAP represents the minimum pressure in the arteries before the next cardiac contraction, providing insight into vascular resistance and vasodilation. MAP, which represents the average pressure throughout the cardiac cycle, is crucial for assessing tissue perfusion, as it is generally closer to DAP due to the longer duration of diastole [2]. Understanding these parameters is essential for evaluating and managing hypotensive and hypertensive conditions effectively [3].

The BP is a critical hemodynamic parameter regularly used to evaluate cardiovascular function and ensure adequate perfusion of organs and tissues [4]. Therefore, BP monitoring is recommended during anesthetic procedures in dogs to optimize cardiovascular stability [5]. Hypotension, defined as an MAP below 65 mmHg or a SAP below 90 mmHg, is a common intraoperative complication in dogs [6]. This condition can lead to cellular hypoxia and ischemic injury to vital organs, as well as an increase in morbidity and mortality rates [7]. Consequently, the early recognition of this condition is crucial for implementing therapeutic interventions to restore normal hemodynamic function [8].

Direct (invasive) BP blood pressure measurement via intra-arterial catheterization is considered the gold standard for BP evaluation. However, limitations such as the requirement for technical expertise and the risks of infection, thrombosis, and hemorrhage make its use more complicated in certain situations [9]. Thus, non-invasive methods like Doppler and oscillometry can be utilized as alternatives to invasive BP (IBP) monitoring during anesthesia [4].

Assessing blood pressure with Doppler devices is a common method for monitoring systolic arterial pressure in anesthetized dogs. However, Doppler has certain limitations, as it doesn’t evaluate MAP or DAP and may exhibit poor agreement when compared to IBP measurement techniques [10]. Similarly, traditional oscillometric devices have exhibited inconsistent performance, particularly in hypotensive states, where accurate BP readings are critical [11].

The introduction of advanced oscillometric devices, specifically those utilizing the linear deflection oscillometry (LDO) algorithm, also known as high-definition oscillometry (HDO), represents a significant advancement in non-invasive BP monitoring [12]. This technique enables real-time recording of the pulse wave curve, providing precise measurements of SAP, MAP, and DAP pressures [13]. By enhancing sensitivity to low-amplitude oscillations, HDO improves the detection of subtle arterial wall vibrations, facilitating more accurate identification of hemodynamic changes. Furthermore, the use of a specialized algorithm for pulse amplitude detection and enhanced artifact recognition, even at high heart rates, increases the precision and reliability of BP measurements. These advancements allow LDO to measure very low pressures, offering improved diagnostic capability, with reduced artifacts and enhanced real-time analysis precision [14].

Although LDO has been used in some studies for monitoring BP in anesthetized dogs [10,15,16] and cats [12,13], the values derived from this method depend significantly on specific algorithms [17]. Hence, introducing a new LDO algorithm requires comparative clinical studies to verify its accuracy and reliability [18]. The purposes of this study were to (1) evaluate the agreement between blood pressure measurements obtained using indirect methods (the new LDO algorithm and Doppler) and the direct method (arterial catheterization) and (2) assess the diagnostic ability of these indirect methods in identifying hypotension induced in dogs anesthetized with sevoflurane. We hypothesized that the LDO method used to monitor BP would demonstrate good agreement and that the LDO device would exhibit strong diagnostic capability in identifying normotensive and hypotensive states in anesthetized dogs.

## 2. Materials and Methods

### 2.1. Animals

A prospective, clinical study was conducted involving eleven healthy male dogs classified as American Society of Anesthesiologists physical status (ASA) 1 [19] from May to October 2024, with a median weight of 18 (11–38) kg, all of which underwent orchiectomy at the Veterinary Hospital of Unit of Training Applied to Research and Extension (UFAPE) Veterinary Intensive Care Unit, São Paulo, Brazil. Animals were determined to be healthy based on physical examination and basic diagnostic testing (hematocrit, platelets, hemoglobin, red blood cells, white blood cells, total plasma protein, and serum biochemistry—urea, creatinine, alkaline phosphatase, gamma-glutamyl transferase). Any animals with an ASA 2 or higher, such as those with pre-existing health conditions or those requiring treatment for chronic illness, were excluded.

Prior consent was obtained from the owners of the animals involved, and approval was granted by the Institutional Ethics Committee on Animal Use (CEUA—protocol number 23117.024823/2024-02).

### 2.2. Anesthetic Management

Food was withheld for eight hours, and there was no water fasting before anesthesia. All animals were premedicated with nalbuphine at a dose of 0.5 mg·kg^−1^ administered intramuscularly (Cristália, São Paulo, Brazil), which provided light sedation to facilitate handling. Fifteen minutes after premedication, the cephalic vein was aseptically catheterized, and a continuous intravenous (IV) infusion of lactated Ringer’s solution (5 mL·kg^−1^.h^−1^, IV) was administered throughout the anesthesia period using an infusion pump (Mindray Syringe SP3, Mindray Animal Medical Technology, Shenzhen, China). Anesthesia was induced using IV propofol (3–5 mg·kg^−1^; Propovan, Cristália) until the desired effect was achieved. Following orotracheal intubation, the animal was connected to the rebreathing circuit of the anesthesia machine (Dräger Primus; Drägerwerk AG & Co., Lübeck, Germany). Anesthesia was maintained with an initial end-tidal sevoflurane (ET_SEVO_) concentration of 2.4%, adjusted as needed to achieve a moderate depth of anesthesia based on clinical signs, such as depressed protective reflexes, rotated eyeballs, and loss of palpebral reflexes, with an inspired oxygen fraction of 30%.

Thereafter, neuromuscular blockade was induced with rocuronium (0.6 mg·kg^−1^ IV; Esmeron, Organon, São Paulo, Brazil) and monitored using a train-of-four (TOF) monitor (Stimpod NMS450, Xavant Technology, Pretoria, South Africa) to evaluate the degree of blockade. Mechanical ventilation was initiated connected to a microprocessor-controlled anesthesia ventilator (Dräger Primus; Drägerwerk AG & Co., Lübeck, Germany). Volume-controlled ventilation (VCV) was applied with a tidal volume of 10 mL·kg^−1^, an inspiratory-to-expiratory ratio of 1:2, and a respiratory rate adjusted to maintain an end-tidal carbon dioxide concentration (ETCO_2_) of 35–45 mmHg.

Heart rate, respiratory rate, pulse oximetry (SpO_2_), and esophageal temperature were monitored using a multiparametric monitor (Philips IntelliVue MX500, Boeblingen, Germany). ET_SEVO_ and ETCO_2_ values were assessed with the gas analyzer integrated into the anesthesia machine (Dräger Primus; Drägerwerk AG & Co., Lübeck, Germany).

### 2.3. Invasive Arterial Blood Pressure Measurement

The animals were positioned in left lateral recumbency on a thermal mattress to maintain normothermia. Following the trichotomy and the antiseptic preparation of the metatarsal region, a 20 gauge, 25 mm cannula (Safelet Nipro, São Paulo, Brazil) catheter was introduced into the dorsal metatarsal artery via percutaneous puncture. This catheter was subsequently connected to an arterial pressure transducer (Truwave individual—Edwards Critical Care Division, USA) through a noncompliant tubing filled with heparinized saline solution (5 UI/mL) and interfaced with a multiparametric monitor (Philips IntelliVue MX500, Boeblingen, Germany) to enable the measurement of SAP, MAP, and DAP. Furthermore, a pressurized bag containing saline solution (300 mmHg per second) was also connected. A relative zero (0 mmHg) value was established before initiating the measurements, with the height of the transducers at the level of the manubrium in anesthetized dogs [20].

The pressure transducers employed for IBP measurement in this study were calibrated in advance against a mercury column to ensure the accuracy and reliability of the recorded values. The precision of the arterial pressure waveform was assessed by evaluating the dynamic response of the arterial catheter tubing and the pressure transducer system [17].

### 2.4. Linear Deflection Oscillometry

**Figure 1:**
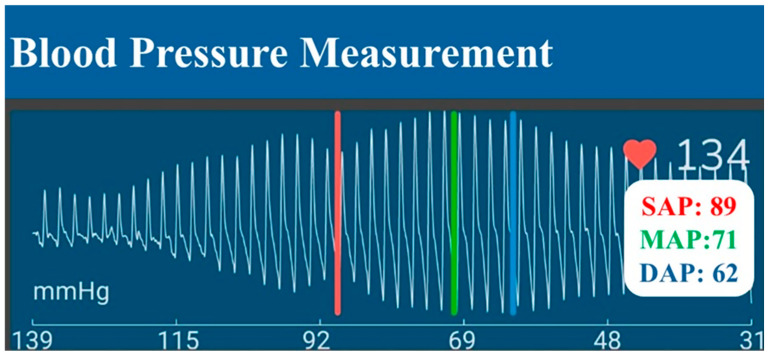
Pulse wave produced by the linear deflection oscillometry (LDO) method. The cuff is first inflated to block arterial flow, then deflated in a controlled, linear manner. As it deflates, blood flow resumes, generating oscillations in the arterial wall and producing pulse waves that are recorded. A dedicated algorithm then processes these pulse waves to determine mean arterial pressure (MAP; green bar), systolic arterial pressure (SAP; red bar), and diastolic arterial pressure (DAP; blue bar).

An InPulse Animal Health LDO (Florianópolis, Brazil) device was utilized to measure BP non-invasively. Cuff size was determined by measuring 30–40% of the left thoracic limb circumference in the mid-third region of the radio-ulnar region using a measuring tape. Subsequently, the cuff was inflated to induce arterial occlusion and was then deflated automatically in a linear manner. As the cuff deflates, blood flow is restored, resulting in oscillations of the arterial wall that enhance the recording of pulse waves. An algorithm subsequently analyses these pulse waves to determine the MAP, SAP, and DAP (Figure 1).

### 2.5. Doppler Ultrasonography

Doppler ultrasonography (Parks Medical Electronics^®^, model 811-B, Aloha, OR, USA) combined with cuffs (InPulse Animal Health, Florianópolis, Brazil) was employed to measure SAP in the animals non-invasively. The cuff was positioned at the same region on the thoracic limb contralateral to the limb used for blood pressure measurement with LDO. The palmar arterial pulse was palpated, and the area was clipped before applying ultrasonic conducting gel. The transducer was placed over the palmar artery between the left thoracic limb’s carpal and metacarpal pads to detect the pulse. The pulse sound was auscultated through headphones connected to the Doppler device. The cuff, attached to the sphygmomanometer (Heine Gamma G7 Small Adult, Optotechnik, Gilching, Deutschland), was inflated gradually until the pulse sound was completely occluded and then slowly deflated until the sound returned, at which point the SAP value was recorded on the manometer.

### 2.6. Experimental Design

After 15 min post-induction, blood pressure was measured using non-invasive (LDO and DU) and invasive methods in a paired approach. Blood pressure agreement was assessed under both normotensive (MAP of 65–80 mmHg) and hypotensive (MAP < 65 mmHg) conditions [6]. Hypotension was induced by increasing the ET_SEVO_. If a concentration greater than 3.6% of ET_SEVO_ was insufficient to induce hypotension, nitroprusside was added (1 μg·kg^−1^·min^−1^, Hypofarma, Ribeirão das Neves, Brazil). Animals were maintained in a hypotensive state for as brief a period as possible. Arterial hypotension was treated immediately after measuring BP by reducing ET_SEVO_ or discontinuing the nitroprusside infusion. If necessary, hypotension was treated by administering a bolus of crystalloids (10 mL·kg^−1^ of lactate Ringer’s solution in 15 min) and an IV bolus of 0.1 mg·kg^−1^ of ephedrine until normalization of MAP; these treatments, if any, were registered accordingly.

Blood pressure measurements obtained through the LDO and Doppler methods were conducted following American College of Veterinary Internal Medicine (ACVIM) guidelines [18,21]. The initial reading from each device was excluded from analysis, and the average of 5 to 7 consecutive consistent values was calculated. To ensure the accuracy and reliability of the data, any readings exhibiting a coefficient of variation (CV) greater than 20% were removed. Subsequently, a stable set of at least three consecutive BP measurements was collected for each hemodynamic state (normotension and hypotension), with the mean of these values utilized for statistical analysis. The LDO and DU device readings were recorded only when the MAP obtained via the invasive technique remained stable, with a variation of less than ±5 mmHg.

After data collection, dogs were submitted to orchiectomy and recovered from anesthesia. Before the initiation of surgery, each dog received an IV dose of meloxicam (0.1 mg·kg^−1^) (Flamavet 0.2%, Agener União, São Paulo, Brazil) and dipyrone (25 mg·kg^−1^) (Analgex V^®^, Agener União, São Paulo, Brazil).

### 2.7. Statistical Analysis

Such data as age, weight, and duration of the procedure were expressed as medians with minimum and maximum values.

The distribution of the residuals was evaluated using the Shapiro–Wilk test. The statistical analysis was conducted using generalized mixed models (GzMM, gamma distribution) or mixed models (MM, linear distribution), with the dependent variables MAP, DAP, and SAP. Fixed effects included the different hemodynamic states (normotension and hypotension) and the methods used to measure blood pressure (direct and indirect). The comparison was made between hemodynamic states within each measurement method and between the different methods within each hemodynamic state. The marginal estimated means with their 95% confidence intervals were expressed, and a significance level of *p* < 0.05 was considered.

The agreement between the direct (IBP) and indirect methods (LDO and Doppler) used for BP monitoring was assessed using the Bland–Altman method for multiple observations per subject [22]. The bias between techniques was determined by calculating the mean difference in BP measurements obtained from direct (IBP) and each indirect method (LDO and Doppler). The 95% limits of agreement (LOAs) were then established as the bias ± (1.96 × standard deviation). A concordance analysis was conducted across all BP ranges and subsequently within the normotensive and hypotensive states. An acceptable level of agreement between the two methods was defined as a mean bias and precision (standard deviation, SD) of less than 10 mmHg and 15 mmHg, respectively. These criteria align with the standards proposed by the ACVIM Hypertension Consensus and Veterinary Blood Pressure Society for validating blood pressure devices in dogs and cats [18].

To determine the sample size required to evaluate diagnostic capability, we must ensure that the LDO effectively predicts hypotension. An area under the receiver operating characteristic (ROC) curve of greater than 0.8 is considered effective. Assuming a null hypothesis of 0.5 (no discrimination between negative and positive for hypotension), a minimum of 10 patients in each hemodynamic state (hypotensive and normotensive) is necessary to detect a difference in the area under the curve of 0.3 with a Type I error of 0.05 and Type II error of 0.2 (test power = 80%). ROC curves were used to assess the ability of the LDO method to detect hypotension, defined as an MAP of less than 65 mmHg by IBP, and using the Doppler method, as an SAP of less than 90 mmHg by IBP. The area under the ROC curve (AUC) was compared, with an AUC of 0.5 indicating no discriminatory ability. The bootstrap method (1000 samples) was used to construct ROC curves, and the Delong method [23] was used to calculate and compare AUCs. The cutoff values were selected based on the highest Youden index, calculated as sensitivity + (specificity-1). Statistical analysis was performed using MedCalc Statistical Software version 20.218 (MedCalc Software bv, Ostend, Belgium; https://www.medcalc.org (accessed on 12 June 2024); 2020) and IBM SPSS Statistics for Windows, version 25 (IBM Corp., Armonk, NY, USA).

## 3. Results

### 3.1. Study Population and Successful Reading

A total of 11 dogs were included in this study, consisting of 3 American bullies, 1 Pitbull, and 7 mixed-breed dogs. The median age of the animals was 4 years (range: 2–6), and the median body weight was 18 kg (range: 11–38). All animals recovered uneventfully from anesthesia, and no postoperative complications were observed until 14 days after anesthesia. The duration of anesthesia, surgery, and recovery were approximately 60 min, 15 min, and 15 min, respectively.

Upon the measurement of blood pressure using indirect methods (Doppler and LDO), the cuffs employed encompassed a range of 34.8% to 38.1% of the limb circumference.

Blood pressure measurements were obtained under both normotensive and hypotensive conditions. One animal did not maintain hemodynamic stability during the hypotensive phase and was evaluated only in the normotensive state. Invasive BP was maintained within the hypotensive range (MAP < 65 mmHg and SAP < 90 mmHg) for a median duration of 15 min (range: 5–20 min). Nitroprusside infusion was utilized in two animals to induce hypotension, to achieve the target MAP and SAP ranges, in conjunction with an increase in sevoflurane concentrations. No treatments other than reducing ET_SEVO_ or discontinuing nitroprusside infusion were necessary to normalize MAP following induced hypotension. Hematomas or other complications related to arterial cannulation were not observed.

In total, 147 measurements were recorded, with 77 in the normotensive state and 70 in the hypotensive state. All measurements were successfully collected using both the LDO and Doppler devices.

**Figure 2:**
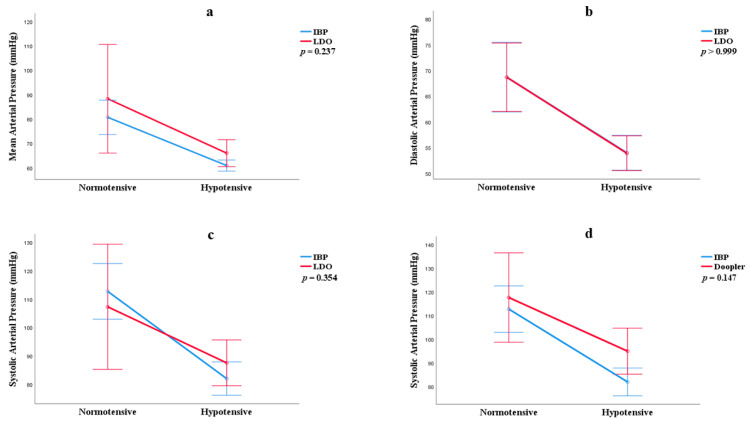
Estimated marginal mean and 95% confidence interval for mean arterial pressure (**a**), diastolic pressure (**b**), and systolic pressure (**c**,**d**) measured using linear deflection oscillometry (LDO) and Doppler compared with IBP measurements. No statistically significant differences were observed between values obtained by indirect methods (Doppler and LDO) and the direct method (IBP), according to GLM analysis with Bonferroni post-hoc testing (*p* > 0.05).

There was no statistical difference between the mean values of MAP (*p* = 0.237), DAP (*p* = 1), and SAP (*p* = 0.354) obtained through direct and indirect methods (Figure 2).

### 3.2. Agreement Between Methods

**Figure 3:**
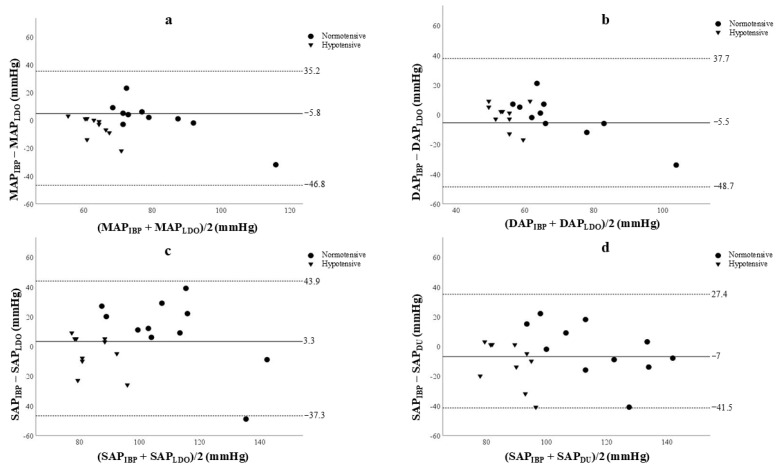
Bland–Altman plot showing the agreement between systolic arterial pressure (SAP), diastolic arterial pressure (DAP), and mean arterial pressure (MAP) measurements obtained using the linear deflection oscillometry (LDO) device (**a**–**c**) or Doppler (DU) device (**d**) compared to the invasive blood pressure (IBP) method. Each point represents a paired comparison (mean of seven measurements per animal) between methods in 11 anesthetized dogs in normotensive (*n* = 11) and hypotensive (*n* = 10) states. Dashed lines indicate the upper and lower limits of agreement, and the solid line represents the mean difference (bias).

The Bland–Altman plots are illustrated in Figure 3. Overall, the values for MAP, DAP, and SAP obtained through LDO were overestimated compared to those derived from the IBP.

Although the biases for the majority of measurements remained consistently below 10 mmHg and the SD was less than 15 mmHg in the hypotensive state, substantial variability in the limits of agreement (LOA) and precision was observed in the normotensive hemodynamic state (Table 1).

The SAP measurements determined by the Doppler method were identified as overestimated during the hypotensive state in relation to those obtained from IBP assessments (Table 2). A supplementary assessment of the agreement analysis between SAP measurements obtained by Doppler and IBP under hypotensive conditions was performed, excluding the two animals requiring nitroprusside for hypotension induction. This subset analysis revealed a bias of −12.42, with LOAs ranging from −46.89 to 22.4 mmHg. These findings demonstrate that the inclusion or exclusion of animals administered nitroprusside did not significantly impact the agreement parameters.

### 3.3. Ability to Detect Hypotension

The AUC was 0.809 (*p* = 0.001) for identifying hypotension (MAP < 65 mmHg) using LDO and 0.798 (*p* = 0.005) for detection of hypotension (SAP < 90 mmHg) with Doppler. An MAP of ≤72 mmHg obtained using LDO was determined to be the most effective cutoff for detecting hypotension, demonstrating a sensitivity of 90% and specificity of 63%. In contrast, for Doppler measurements, the optimal threshold was established at an SAP of ≤ 100 mmHg, which exhibited a sensitivity of 77.8% and specificity of 81.8% in identifying hypotension (Table 3).

## 4. Discussion

This study compared the concordance and diagnostic accuracy of two non-invasive blood pressure measurement methods, LDO and Doppler, with the IBP method in anesthetized dogs. The results indicated that LDO demonstrated better concordance with IBP, particularly under hypotensive conditions. These findings underscore the clinical potential of LDO as a viable alternative to invasive monitoring, particularly in situations where access to invasive methods is limited or impractical. Although the LDO has been employed in previous studies with anesthetized animals [10,12,15,16], to the authors’ knowledge, this study is the first to precisely evaluate the Inpulse algorithm for blood pressure measurement and assess the diagnostic capability of the LDO in detecting hypotension in anesthetized dogs. This algorithm was designed to optimize sensitivity in detecting arterial oscillations, particularly in challenging hemodynamic scenarios such as hypotension. This technical advancement is significant, as conventional oscillometric techniques often show limitations, especially in hypotensive states [11]. Thus, using the LDO may provide a new perspective on the applicability of non-invasive methods for monitoring in anesthesia and critical states.

Blood pressure measurement using non-invasive methods was successful in all cases, which is consistent with previous studies [10,12,16]. This finding supports that non-invasive methods are particularly advantageous in anesthetized dogs, enabling continuous monitoring without the complications associated with invasive techniques [24].

The Bland–Altman analysis between LDO and IBP measurements for MAP and DAP revealed considerable variability between methods, particularly in the normotensive state. Previous studies in anesthetized dogs and rabbits [10,25] have also documented limitations in the precision of LDO compared to the invasive standard. Our study observed that while the LDO’s bias remains acceptable within the normotensive range, it demonstrates significant variability due to wide LOAs in MAP and DAP measurements. However, in hypotensive states, the LDO demonstrated significantly better concordance with the invasive method, meeting the bias and precision criteria set by the ACVIM [18]. This suggests that the LDO can be a valuable and reliable tool for clinical monitoring and immediate therapeutic support in critical hemodynamic states, such as hypotension. However, its use in normotension should be approached cautiously due to such wide LOAs. Furthermore, the accurate measurement of diastolic pressure is crucial for assessing tissue perfusion and vascular resistance, being a fundamental determinant of coronary blood flow [26] reinforcing the LDO’s value in managing critically ill or anesthetized patients.

In relation to SAP, Doppler and LDO overestimate blood pressure values, particularly in hypotensive patients, compared to the IBP method. Furthermore, Doppler does not meet the criteria established by the ACVIM, as observed by Seliskar et al. [10]. These findings indicate that although Doppler is widely used, SAP measurements in hypotensive conditions should be interpreted with caution. Overestimation may lead to an underestimation of hypotension severity or delayed recognition of the condition. Additionally, since Doppler only measures systolic pressure, it is limited in its main utility during anesthesia and monitoring of the critical ill patients, especially considering that SAP is known to be the most variable across all measurement methods [10,11]. This variability can be particularly detrimental to patient care and may obscure the actual hemodynamic status.

While this study demonstrated a good agreement between the MAP values obtained through LDO, the presence of an LOA exceeding 10 mmHg and the overestimation of SAP values by Doppler in hypotensive animals may have significant clinical implications. These include delayed therapeutic interventions for hypotension or underestimation of its severity, which could adversely affect patient management during anesthesia.

This discrepancy raises concerns regarding identifying hypotensive patients when employing the same reference value established by the IBP method. Therefore, to enhance the diagnostic accuracy of the LDO and Doppler techniques in detecting hypotension in anesthetized dogs, the optimal cutoff point was determined through ROC curve analysis.

Typically, ROC curves with areas exceeding 0.9 are considered excellent, while those with values between 0.8 and 0.9 are classified as very good [27]. The AUC values obtained in this study for both LDO and Doppler reflect good diagnostic capability for both methods. The LDO demonstrated slightly superior performance in identifying hypotension, particularly for MAP, resulting in an adequate positive predictive value (PPV) and negative predictive value (NPV). These findings suggest that the LDO is effective in both identifying and excluding the hypotensive state in anesthetized dogs. In contrast, although the Doppler’s PPV was higher than that of the LDO, its lower NPV indicates that while effective in confirming hypotension, the Doppler may be less effective in excluding this condition. This distinction is clinically relevant, as a high NPV is crucial to minimize the occurrence of false negatives in anesthetized patients [28].

Thus, the LDO may prove more suitable for continuous real-time monitoring of MAP and DAP, allowing for rapid interventions when necessary. On the other hand, the Doppler can serve as a confirmation method in cases where hypotension is suspected but not confirmed. Furthermore, it is essential to emphasize that the interpretation of the results must consider both the clinical and hemodynamic presentation of the patient and the inherent limitations of each method. Therefore, employing multiple monitoring approaches may represent the most effective strategy for ensuring improved prognoses during the anesthetic period.

This study presents certain limitations. First, the LDO device was not tested in animals under 5 kg, where smaller arterial sizes may impede accurate pulse wave recognition [17]. The study also did not evaluate the ability of this new technology to detect and monitor blood pressure trends across diverse clinical conditions, particularly in awake animals and those in a state of hypertension. Future research is needed to assess this device under fluctuating blood pressure ranges, in animals weighing less than 5 kg, and in cats. Additionally, real clinical studies would provide further insights into the practical applications of this method.

## 5. Conclusions

In conclusion, although the observed biases fall within the limits recommended by the ACVIM, the wide LOAs and elevated standard deviations (precision) suggest limited concordance between the LDO and IBP. Conversely, the Doppler method tends to overestimate SAP values, particularly in hypotensive conditions, and does not provide measurements for MAP and DAP, which are critical for comprehensive hemodynamic assessment. Both indirect methods exhibit a moderate ability to identify hypotension; however, the LDO is characterized by greater sensitivity while demonstrating lower specificity relative to the Doppler.

## Figures and Tables

**Table 1:** Agreement of blood pressures in anesthetized dogs measured by the invasive arterial blood pressure (IBP) technique and linear deflection oscillometry (LDO) device.

Parameter	Hemodynamic State	Bias (mmHg)	Precision (mmHg)	LOA (mmHg)	≤±10 mmHg	≤±20 mmHg
SAP	Overall	3.3	20.7	−37.3–43.9	52.4%	66.67%
	Normotension	10.6	23.6	−35.7–57	27%	54.5%
	Hypotension	−5.6	12.5	−30.1–19	77.7%	77.7%
MAP	Overall	−5.8	20.9	−46.8–35.2	71.42%	80.95%
	Normotension	−6.4	28.8	−62.4–49.7	63.63%	72.7%
	Hypotension	−5.1	7.9	−20.7–10.5	80%	90%
DAP	Overall	−5.5	22	−48.7–37.7	71.42%	85.71%
	Normotension	−9.7	29.4	−67.4–47.9	60%	70%
	Hypotension	−0.8	8.62	−17.7–16.1	80%	100%

Interpretation: SAP: systolic arterial pressure; MAP: mean arterial pressure; DAP: diastolic arterial pressure; Bias: mean of difference (IBP—LDO); Precision: standard deviation (SD) of the bias; LOA: limits of agreement (mean bias ± 1.96 × SD); percentages of LDO measurements (SAP, DAP and MAP) lying within ≤±10 or 20 mmHg of the corresponding IBP values. The overall analysis includes all measured value pairs, whereas the normotension and hypotension categories comprise measurement pairs with an MAP of 65–90 mmHg and an MAP ≤ 65 mmHg, respectively, using the IBP method.

**Table 2:** Agreement of blood pressures in anesthetized dogs measured by the invasive arterial blood pressure (IBP) technique and Doppler (DU) device.

Parameter	Hemodynamic State	Bias (mmHg)	Precision (mmHg)	LOA (mmHg)	<±10 mmHg	<±20 mmHg
SAP	Overall	−7	17.55	−41.5–27.4	47.6%	80.9%
	Normotension	−2.5	17.9	−37.7–32.6	36.4%	81.8%
	Hypotension	−13	15.45	−43.3–17.3	60%	80%

Interpretation: SAP: systolic arterial pressure; Bias: mean of difference (IBP—DU); Precision: standard deviation (SD) of the bias; LOA: limits of agreement (mean bias ± 1.96 × SD); percentages of DU measurements lying within ≤±10 or 20 mmHg of the corresponding IBP values. The overall analysis includes all measured value pairs, whereas the normotension and hypotension categories comprise measurement pairs with an SAP ≥ 90 mmHg and an SAP ≤ 90 mmHg, respectively, using the IBP method.

**Table 3:** Areas under the receiver operating characteristic curve (AUC) and optimal diagnostic cutoffs of detection. Hypotension^*^ by linear deflection oscillometry (LDO) or Doppler device (DU).

	AUC	Cutoff	Se	Sp	PPV	NPV
MAP_LDO_	0.809 (0.556–0.945)	≤72 mmHg	90 (55.5–99.7)	63.6 (30.8–89.1)	69.2 (50.1–83.5)	87.5 (50.8–97.9)
		≤65 mmHg	50 (18.7–81.3)	82 (48.2–97.7)	71.4 (38.2–91)	64.3 (47.7–78)
SAP_DU_	0.798 (0.520–0.960)	≤100 mmHg	77.8 (40–97.2)	81.8 (48.2–97.7)	77.8 (48.8–92.8)	81.8 (56.2–94)
		≤90 mmHg	44.4 (13.7–78.8)	81.8 (48.2–97.7)	66.7 (31.9–89.5)	64.3 (48.5–77.5)

Interpretation: AUC calculated for systolic arterial pressure (SAP) < 90 mmHg or mean arterial pressure (MAP) < 65 mmHg by IBP. Se, sensitivity; Sp, specificity; PPV, positive predictive value; NPV, negative predictive. Data are expressed as a percentage (95% confidential interval).

## Data Availability

The datasets used and analysed during the current study are available from the corresponding author on reasonable request.
